# Point Shear Wave Elastography for Splenic Assessment: A Comparative Study of Splenomegaly and Normal Spleens

**DOI:** 10.7759/cureus.62869

**Published:** 2024-06-21

**Authors:** Parag V Patil, Saksham Jain

**Affiliations:** 1 Radiodiagnosis, Dr. D. Y. Patil Medical College, Hospital and Research Centre, Dr. D. Y. Patil Vidyapeeth, Pune, IND

**Keywords:** ultrasound elastography, spleen elastography, splenomegaly, spleen stiffness, point shear wave elastography

## Abstract

Background

The spleen, a key immunological organ, plays a crucial role in filtering aged or damaged red blood cells. Splenomegaly, an enlargement of the spleen, can arise from a variety of physiological and pathological conditions, including hematological disorders, hepatic diseases, and infections. Accurate diagnosis and evaluation of splenomegaly are essential for effective treatment.

Objectives

The objective of this study was to evaluate splenic stiffness in patients with splenomegaly using point shear wave elastography (pSWE) and compare the findings with those of individuals with normal spleen.

Materials and methods

This hospital-based observational study was conducted at Dr. D. Y. Patil Medical College, Hospital and Research Centre, Pune, India, from October 2022 to December 2023. The study included 56 participants, divided into two groups: 28 patients with splenomegaly and 28 healthy controls. Spleen stiffness was measured using a Samsung HS70A ultrasound machine (Samsung Electronics Pvt. Ltd., Seoul, South Korea), and pSWE was performed with a curvilinear probe. Data were analyzed using IBM SPSS Statistics for Windows, Version 26.0 (Released 2019; IBM Corp., Armonk, New York, United States), and the significance of differences was assessed using an independent t-test with a p-value of <0.05, considered statistically significant.

Results

The mean spleen stiffness, measured in kilopascals (kPa), was significantly higher in individuals with splenomegaly (32.05 ± 12.54 kPa) compared to controls (22.9 ± 9.49 kPa) (p = 0.003). A positive correlation (rho = 0.482, p < 0.001) was observed between spleen diameter and spleen stiffness.

Conclusion

This study demonstrates that pSWE is an effective, non-invasive tool for assessing spleen stiffness. The higher spleen stiffness in splenomegaly patients underscores pSWE's diagnostic utility, with a positive correlation between spleen diameter and stiffness. Further multi-center studies are recommended to validate these findings, highlighting pSWE's promise in evaluating and monitoring splenic disorders.

## Introduction

The spleen is a principal immunological organ in the human body, tasked with the elimination of aged or impaired red blood cells from the circulatory system. The average size of the spleen in adults, as measured by ultrasonography, is typically less than 13 cm in length and 5 cm in thickness [1].

Splenomegaly is a frequently observed disorder in clinical settings. It can result from physiological conditions, such as recovery following blood loss or pregnancy, but it is also a consequence of infection or various chronic medical conditions. Causes of splenomegaly can be broadly divided into hematological disorders, hepatic diseases, and infectious diseases [2,3]. Hematological disorders encompass a range of conditions, such as myeloproliferative disorders, leukemia, lymphoma, and hemolytic disorders. Hepatic pathologies that can lead to splenomegaly include cirrhosis, portal hypertension, portal vein thrombosis, and malignancy. Additionally, infections, such as malaria, typhoid, cytomegalovirus, and acute mononucleosis, can cause splenomegaly [2,3]. Identifying the cause of splenomegaly is crucial for effective therapy, as treatment focuses on addressing the underlying cause [3].

Elastography is a novel diagnostic technique used to measure the rigidity (elasticity) of soft tissues [4]. Its utility stems from the observation that diseased tissues typically exhibit greater rigidity and reduced elasticity compared to adjacent healthy tissues. Furthermore, there is a consensus that elasticity undergoes the most rapid alterations in pathologically changed tissue compared to other parameters [5].

Elastography can determine the mechanical characteristics of tissues completely non-invasively. The four main elastographic techniques are magnetic resonance elastography (MRE), two-dimensional shear wave elastography (2D-SWE), point shear wave elastography (pSWE), and transient elastography (TE). TE utilizes a 3.5-MHz ultrasonic transducer connected to a low-amplitude vibrator. This combination produces low-frequency shear waves, which are then used to detect the velocity of the waves as they pass through the tissue. This measurement helps evaluate the stiffness of the tissue. pSWE utilizes ultrasound tracking beams with a solitary push beam to produce shear waves within a specific region of interest (ROI) to measure tissue stiffness. 2D-SWE employs focused ultrasonic beams to generate radiation force, which is then used to measure shear waves in real time. These shear waves are utilized to construct a two-dimensional color map that represents tissue elasticity [6]. In shear-wave elastography, stiffness values result from elastic return forces acting on a tissue against a shear force, which is the deformation force. Shear-wave elastography uses ultrasound-induced acoustic radiation to measure the velocity of shear waves, providing information about the mechanical characteristics of tissues [7]. The theory behind elastography is that certain disorders cause tissues to become "harder" than healthy tissues; the higher the shear wave velocity, the stiffer the tissue [8].

The objective of the study was to use pSWE to measure splenic stiffness in patients with splenomegaly and individuals with normal spleen size. The study will then compare the results between the two groups.

## Materials and methods

This hospital-based observational study was performed at Dr. D. Y. Patil Medical College, Hospital and Research Centre in Pune, India, from October 2022 to December 2023. The study comprised 56 participants. The Institutional Ethics Subcommittee of Dr. D. Y. Patil Medical College, Hospital and Research Centre approved this study (Reference number: I.E.S.C/420/2022).

Inclusion and exclusion criteria

The following inclusion criteria were used: (i) Participants must be older than 18 years; (ii) for the cases group, patients referred to the radiology department with splenomegaly (maximum length >13 cm on ultrasound); and (iii) for the control group, healthy individuals without a history of substantial alcohol consumption (less than 30 g/day for men and less than 20 g/day for women), systemic diseases, metabolic diseases, or any other diseases affecting the spleen. Patients unable to hold their breath in a neutral position, with gross ascites, pregnant individuals, and those with a splenic mass were not included in the study.

Technique

A Samsung HS70A ultrasound machine (Samsung Electronics Pvt. Ltd., Seoul, South Korea) was used for the ultrasound examinations.

1. The longitudinal spleen diameter was recorded at the splenic hilum for each individual while they lay supine, using an ultrasound probe positioned craniocaudally along the intercostal spaces to measure the greatest distance between the spleen's superomedial and inferolateral points.

2. pSWE imaging was performed on all individuals utilizing a curvilinear probe.

3. Individuals were scanned in the right lateral decubitus position, with their left arm fully extended away from their body.

4. Individuals were instructed to hold their breath in a neutral position.

5. In every individual, the stiffness of the spleen was assessed by pSWE at a depth of 1 to 2 cm below the capsule of the spleen.

6. The ROI was devoid of visible vessels.

7. Spleen stiffness was assessed by conducting examinations at three specific locations: the upper pole, hilum, and lower pole, using the left intercostal and subcostal approaches.

8. Three measurements were recorded at each location of the spleen.

9. For every individual, the median values were determined by averaging nine successful measurements and were reported in kilopascals (kPa).

**Figure 1:**
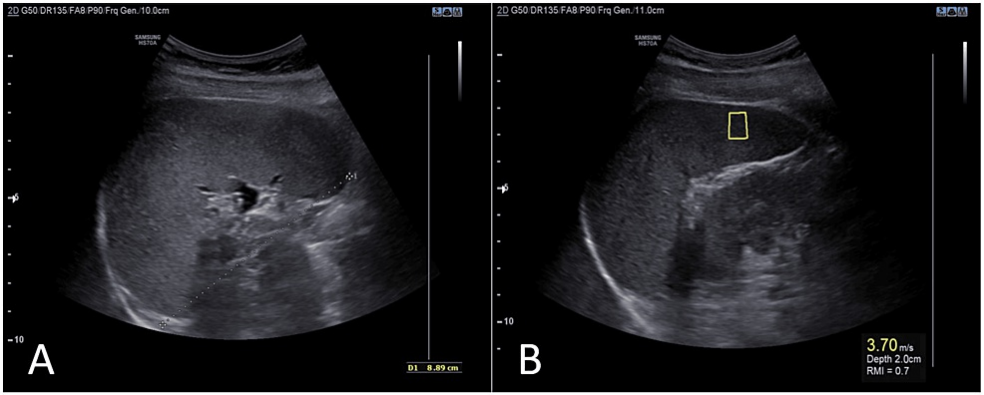
B-mode ultrasound image showing longitudinal spleen diameter measurement and pSWE image showing the region of interest (ROI) B-mode ultrasound image showing the longitudinal spleen measurement (dotted line) (A) and point shear wave elastography depicting the ROI (yellow box) (B).

Figure 1 displays a B-mode ultrasound image demonstrating the longitudinal spleen diameter measurement and a pSWE image showing the ROI.

Statistical analysis

Data were entered into Microsoft Excel (Microsoft Corporation, Redmond, Washington, United States). The analysis was conducted using IBM SPSS Statistics for Windows, Version 26.0 (Released 2019; IBM Corp., Armonk, New York, United States). Categorical variables were expressed in frequency and proportions. The mean (SD) and the median (IQR) were calculated for the continuous variables. Spleen stiffness was found to be normally distributed. An independent t-test was applied to test the significance of the difference in spleen stiffness between the cases and controls. A p-value of <0.05 was considered statistically significant.

## Results

Study population

The study comprised a total of 56 participants, who were separated into two groups: 28 patients diagnosed with splenomegaly (cases) and 28 individuals with spleens of normal size (controls). Each group consisted of 50% of the entire research population.

The age range of individuals in the splenomegaly group was 28 to 59 years, with a mean age of 42.93 years (±9.27 years). The control group comprised individuals aged 22 to 65 years, with a mean age of 39.50 years (±10.30 years). The median age was 42 years for the cases and 41 years for the controls. This suggests that the cases exhibit a slightly higher mean age compared to the controls.

Table 1 shows the descriptive statistics of age for both cases and controls.

**Table 1:** Descriptive statistics of age for both cases and controls

Statistic	Cases	Controls
Mean age (years)	42.93	39.50
Median age (years)	42	41
Standard deviation	9.27	10.30

In terms of sex distribution, the splenomegaly group included 13 females (46.4%) and 15 males (53.6%). The control group had an equal distribution of males and females: 14 females (50%) and 14 males (50%). Overall, the study population consisted of 27 females (48.2%) and 29 males (51.8%).

Table 2 shows the sex distribution for both cases and controls.

**Table 2:** Sex distribution for both cases and controls

Sex	Group	Frequency (out of 56)	Percentage
Males	Cases	15	53.6
	Controls	14	50
	Total	29	51.8
Females	Cases	13	46.4
	Controls	14	50
	Total	27	48.2

Longitudinal spleen diameter

The longitudinal spleen diameter was measured in both groups. In the splenomegaly group, the spleen diameter ranged from 13.0 cm to 20.0 cm, with a mean diameter of 15.1 cm (±1.7 cm). In the control group, the spleen diameter ranged from 8.1 cm to 11.9 cm, with a mean diameter of 9.7 cm (±1.1 cm).

Causes of splenomegaly

The causes of splenomegaly among the patients were diverse. The commonest cause was chronic liver disease, which accounted for 11 cases (39.3%). Dengue fever was identified as the cause in seven cases (25.0%). Other causes included megaloblastic anemia in three cases (10.7%) and hemolytic anemia in two cases (7.1%). Less common causes included iron deficiency anemia, malaria, chronic myeloid leukemia, portal vein thrombosis, and typhoid, each responsible for one case (3.6%).

Table 3 shows the various causes of splenomegaly among the cases.

**Table 3:** Causes of splenomegaly among the cases

Cause	Frequency (out of 28)	Percentage
Chronic liver disease	11	39.3
Dengue	7	25.0
Megaloblastic anemia	3	10.7
Hemolytic anemia	2	7.1
Iron deficiency anemia	1	3.6
Malaria	1	3.6
Chronic myeloid leukemia	1	3.6
Portal vein thrombosis	1	3.6
Typhoid	1	3.6

Spleen stiffness

Spleen stiffness was measured using pSWE in both groups. The mean spleen stiffness in the splenomegaly group was 32.05 kPa (±12.54 kPa), with values ranging from 10.7 kPa to 52.9 kPa. In the control group, the mean spleen stiffness was 22.9 kPa (±9.49 kPa), with values ranging from 8.8 kPa to 44.3 kPa.

The analysis of spleen stiffness between patients with splenomegaly (cases) and normal individuals (controls) revealed significant differences. The mean spleen stiffness was 32.05 kPa for cases and 22.90 kPa for controls, with median values of 33.50 kPa and 20.40 kPa, respectively. The standard deviations were 12.54 kPa for cases and 9.50 kPa for controls. An independent sample t-test was performed to assess the statistical significance of the difference in mean stiffness between the two groups. The results showed a t-statistic of 3.078 and a p-value of 0.0033, indicating a statistically significant difference (p < 0.05). Thus, the null hypothesis that there is no difference in spleen stiffness between cases and controls was rejected. These findings demonstrate that mean spleen stiffness is significantly higher in patients with splenomegaly than in normal individuals.

**Figure 2:**
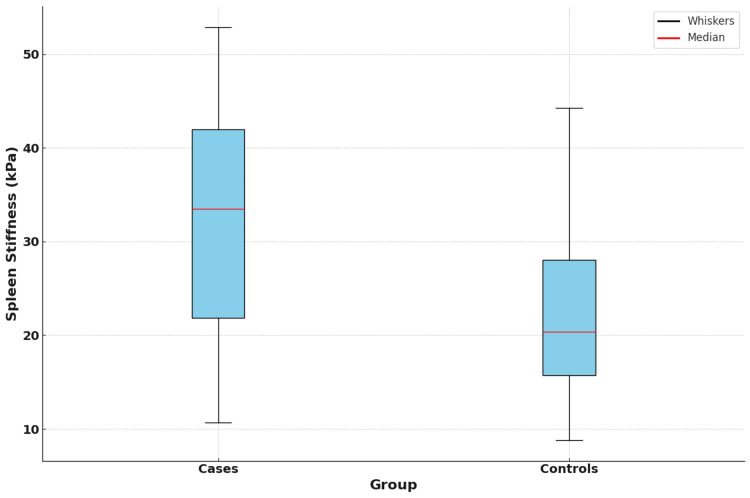
Box and whisker plot comparing spleen stiffness between cases and controls The box and whisker plot demonstrates that cases (patients with splenomegaly) exhibit higher median spleen stiffness values and a broader range compared to controls (patients without splenomegaly). The blue boxes represent the interquartile range (IQR) of spleen stiffness for each group, with the red lines indicating the median values. The black whiskers extend to the minimum and maximum values that fall within 1.5 times the IQR from the first and third quartiles.

Figure 2 shows a box and whisker plot comparing spleen stiffness between cases and controls.

Correlation between longitudinal spleen diameter and spleen stiffness

The Spearman correlation analysis between spleen diameter and spleen stiffness revealed a moderate positive correlation with a rho (ρ) of 0.482 and a statistically significant p-value of 0.00017. This indicates that there is a meaningful relationship between these two variables, where an increase in spleen diameter generally corresponds to an increase in spleen stiffness. The statistical significance of the p-value suggests that this observed correlation is unlikely to be due to random chance, adding confidence to the finding. This relationship may provide important insights into the pathophysiology of splenomegaly and its impact on spleen stiffness, potentially aiding in the clinical assessment and management of patients with spleen-related disorders.

**Figure 3:**
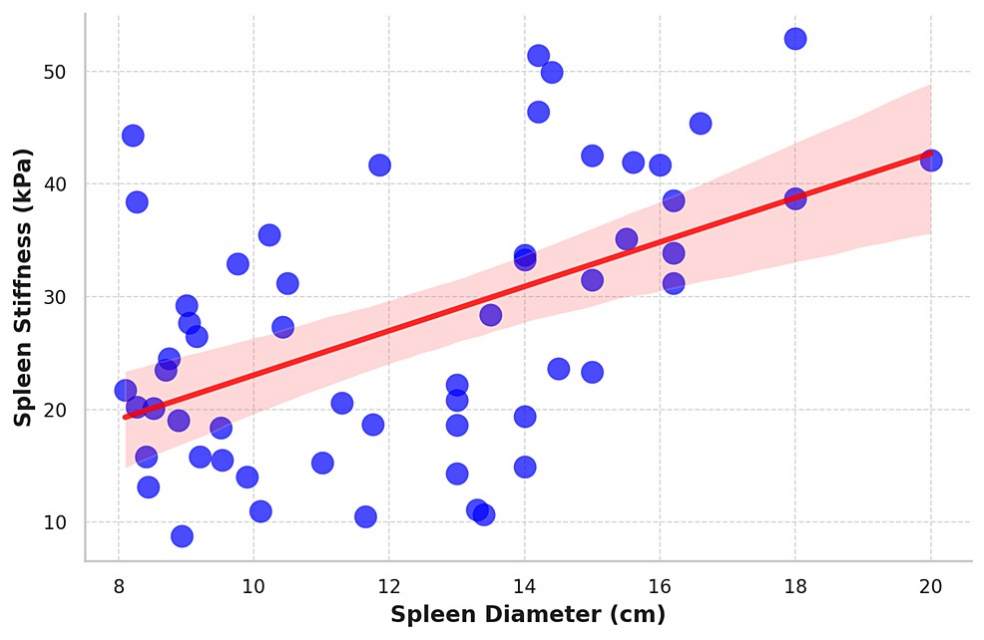
Scatter plot showing the correlation between spleen stiffness and longitudinal spleen diameter The scatter plot shows a positive correlation between longitudinal spleen diameter (cm) and spleen stiffness (kPa), with individual data points represented as blue dots. The red line indicates the linear regression, while the shaded area represents the 95% confidence interval around the regression line.

Figure 3 shows a scatter plot showing the correlation between spleen stiffness and longitudinal spleen diameter.

## Discussion

There are several causes of splenomegaly because it is linked to several pathophysiological mechanisms and illnesses [9]. Three pathophysiological mechanisms are usually considered to cause splenomegaly: congestion, infiltrative disorders, and lymphoid hyperplasia, also known as reticuloendothelial system hyperplasia [10]. A study conducted by O'Reilly RA [11] classified the causes of splenomegaly into six diagnostic groups: hepatic diseases (36%), hematologic disorders (35%), infectious diseases (16%), inflammatory conditions (5%), primary splenic disorders (4%), and other causes (3%).

The exact mechanisms that cause splenomegaly are not yet fully understood. However, it is likely that in patients with hepatic disease, splenomegaly is caused by congestion from increased blood flow, pulp hyperplasia, and fibrosis [12,13]. Splenomegaly in patients with hematologic disorders is mostly caused by the gradual rise in spleen size and blood flow, increased red blood cell count, enlargement of the red pulp, and expansion of reticular components [14]. Chronic systemic infections, viral illnesses, and immune-mediated disorders can lead to splenomegaly through follicular hyperplasia [3].

Ultrasonography is a cost-effective and dependable technique for diagnosing splenomegaly; however, it cannot identify the cause of splenomegaly; elastography can assist in identifying the origin of splenomegaly in patients [15]. Due to tissue hyperplasia, fibrosis, and splenic congestion, people with enlarged spleens have differing spleen densities. Elastography can be used to quantify the mechanical properties of changes in the spleen [16,17]. Batur et al. (2019) [18] and Yalçın and Demir (2020) [19] have tried to differentiate three main categories (hepatoportal, myeloproliferative, and infectious) of causes of splenomegaly based on the measurement of splenic stiffness.

Our study found that the mean spleen stiffness value in patients with splenomegaly was significantly higher than that of individuals with normal spleen size. The mean spleen stiffness in patients with splenomegaly was 32.05 ± 12.54 kPa, compared to 22.9 ± 9.49 kPa in those without splenomegaly (p = 0.003). These results are consistent with the findings of Cho et al. (2018) [20], who reported a mean spleen stiffness of 35.1 ± 14.4 kPa in patients with splenomegaly and 23.7 ± 8.7 kPa in those without splenomegaly (p < 0.001). The slight variations between our findings and those of Cho et al. might be attributed to differences in patient demographics and the elastography techniques used. Nonetheless, this similarity in findings reinforces the reliability of pSWE as a diagnostic tool for assessing spleen stiffness.

The significantly higher spleen stiffness in patients with splenomegaly compared to those without splenomegaly was also concluded in a study by Batur et al. (2019) [18], which reported similar findings. Their study found a significant difference in spleen stiffness values between the two groups, with a p-value of 0.001. This further supports the consistency and reliability of using spleen stiffness measurements in clinical practice. The agreement between our findings and those of Batur et al. highlights the potential of pSWE as a non-invasive diagnostic tool.

There were a few limitations to our study. First, the study was carried out at a solitary center, perhaps leading to the introduction of selection bias. Furthermore, the limited sample size hindered the ability to extrapolate the findings to the wider community. Finally, a single radiologist conducted all measurements, and the study did not evaluate the rates of agreement between different observers.

The studies on shear wave elastography (SWE) of the spleen highlight its significant clinical potential. Karagiannakis et al. (2019) [21] demonstrated that 2D-SWE could effectively exclude high-risk varices in patients with liver cirrhosis, potentially reducing the need for upper gastrointestinal endoscopy. Hirooka et al. (2022) [22] showed a strong correlation between splenic shear wave speed and hepatic venous pressure gradient, identifying it as a useful marker for predicting high-risk varices in patients with chronic liver disease. Collectively, these findings suggest that SWE of the spleen can enhance diagnostic accuracy, minimize invasive procedures, and provide critical information on disease etiology and progression.

## Conclusions

This study demonstrates that pSWE is an effective, non-invasive tool for measuring spleen stiffness. The higher spleen stiffness in splenomegaly patients underscores pSWE's diagnostic utility. Additionally, there was a positive correlation between spleen diameter and stiffness. Further multi-center studies are recommended to validate these results. In conclusion, pSWE shows significant promise in improving the evaluation and monitoring of splenic disorders.

## References

[1] Ishibashi H, Higuchi N, Shimamura R, Hirata Y, Kudo J, Niho Y (1991). Sonographic assessment and grading of spleen size. J Clin Ultrasound.

[2] Curovic Rotbain E, Lund Hansen D, Schaffalitzky de Muckadell O, Wibrand F, Meldgaard Lund A, Frederiksen H (2017). Splenomegaly - diagnostic validity, work-up, and underlying causes. PLoS One.

[3] McKenzie CV, Colonne CK, Yeo JH, Fraser ST (2018). Splenomegaly: pathophysiological bases and therapeutic options. Int J Biochem Cell Biol.

[4] Bamber J, Cosgrove D, Dietrich CF (2013). EFSUMB guidelines and recommendations on the clinical use of ultrasound elastography. Part 1: basic principles and technology. Ultraschall Med.

[5] Ophir J, Céspedes I, Ponnekanti H, Yazdi Y, Li X (1991). Elastography: a quantitative method for imaging the elasticity of biological tissues. Ultrason Imaging.

[6] Yu JH, Lee HA, Kim SU (2023). Noninvasive imaging biomarkers for liver fibrosis in nonalcoholic fatty liver disease: current and future. Clin Mol Hepatol.

[7] Dietrich CF, Bamber J, Berzigotti A (2017). EFSUMB guidelines and recommendations on the clinical use of liver ultrasound elastography, update 2017 (long version). Ultraschall Med.

[8] Nightingale K (2011). Acoustic radiation force impulse (ARFI) imaging: a review. Curr Med Imaging Rev.

[9] Sjoberg BP, Menias CO, Lubner MG, Mellnick VM, Pickhardt PJ (2018). Splenomegaly: a combined clinical and radiologic approach to the differential diagnosis. Gastroenterol Clin North Am.

[10] Pozo AL, Godfrey EM, Bowles KM (2009). Splenomegaly: investigation, diagnosis and management. Blood Rev.

[11] O'Reilly RA (1996). Splenomegaly at a United States County Hospital: diagnostic evaluation of 170 patients. Am J Med Sci.

[12] Furuichi Y, Moriyasu F, Taira J (2013). Noninvasive diagnostic method for idiopathic portal hypertension based on measurements of liver and spleen stiffness by ARFI elastography. J Gastroenterol.

[13] Hirooka M, Ochi H, Koizumi Y (2011). Splenic elasticity measured with real-time tissue elastography is a marker of portal hypertension. Radiology.

[14] Fraquelli M, Giunta M, Pozzi R (2014). Feasibility and reproducibility of spleen transient elastography and its role in combination with liver transient elastography for predicting the severity of chronic viral hepatitis. J Viral Hepat.

[15] Iannitto E, Tripodo C (2011). How I diagnose and treat splenic lymphomas. Blood.

[16] Ye XP, Ran HT, Cheng J, Zhu YF, Zhang DZ, Zhang P, Zheng YY (2012). Liver and spleen stiffness measured by acoustic radiation force impulse elastography for noninvasive assessment of liver fibrosis and esophageal varices in patients with chronic hepatitis B. J Ultrasound Med.

[17] Stefanescu H, Grigorescu M, Lupsor M, Procopet B, Maniu A, Badea R (2011). Spleen stiffness measurement using Fibroscan for the noninvasive assessment of esophageal varices in liver cirrhosis patients. J Gastroenterol Hepatol.

[18] Batur A, Alagoz S, Durmaz F, Baran AI, Ekinci O (2019). Measurement of spleen stiffness by shear-wave elastography for prediction of splenomegaly etiology. Ultrasound Q.

[19] Yalçın K, Demir BÇ (2021). Spleen stiffness measurement by shear wave elastography using acoustic radiation force impulse in predicting the etiology of splenomegaly. Abdom Radiol (NY).

[20] Cho YS, Lim S, Kim Y, Sohn JH, Jeong JY (2019). Spleen stiffness measurement using 2‐dimensional shear wave elastography: the predictors of measurability and the normal spleen stiffness value. J Ultrasound Med.

[21] Karagiannakis DS, Voulgaris T, Koureta E, Chloupi E, Papatheodoridis GV, Vlachogiannakos J (2019). Role of spleen stiffness measurement by 2D-shear wave elastography in ruling out the presence of high-risk varices in cirrhotic patients. Dig Dis Sci.

[22] Hirooka M, Koizumi Y, Nakamura Y (2023). Spleen stiffness in patients with chronic liver disease evaluated by 2-D shear wave elastography with ultrasound multiparametric imaging. Hepatol Res.

